# PML2‐mediated thread‐like nuclear bodies mark late senescence in Hutchinson–Gilford progeria syndrome

**DOI:** 10.1111/acel.13147

**Published:** 2020-04-29

**Authors:** Ming Wang, Lulu Wang, Minxian Qian, Xiaolong Tang, Zuojun Liu, Yiwei Lai, Ying Ao, Yinghua Huang, Yuan Meng, Lei Shi, Linyuan Peng, Xinyue Cao, Zimei Wang, Baoming Qin, Baohua Liu

**Affiliations:** ^1^ Shenzhen Key Laboratory for Systemic Aging and Intervention (SAI) National Engineering Research Center for Biotechnology (Shenzhen) Medical Research Center Shenzhen University Health Science Center Shenzhen China; ^2^ Guangdong Key Laboratory for Genome Stability and Human Disease Prevention Department of Biochemistry & Molecular Biology School of Basic Medical Sciences Shenzhen University Health Science Center Shenzhen China; ^3^ Guangdong Provincial Key Laboratory of Stem Cell and Regenerative Medicine Guangzhou Institutes of Biomedicine and Health Chinese Academy of Sciences Guangzhou China; ^4^ Carson International Cancer Center Shenzhen University Health Science Center Shenzhen China; ^5^ Guangdong Provincial Key Laboratory of Regional Immunity and Diseases School of Basic Medical Sciences Shenzhen University Health Science Center Shenzhen China

**Keywords:** HGPS, PML2, senescence, thread‐like PML NBs

## Abstract

Progerin accumulation disrupts nuclear lamina integrity and causes nuclear structure abnormalities, leading to premature aging, that is, Hutchinson–Gilford progeria syndrome (HGPS). The roles of nuclear subcompartments, such as PML nuclear bodies (PML NBs), in HGPS pathogenesis, are unclear. Here, we show that classical dot‐like PML NBs are reorganized into thread‐like structures in HGPS patient fibroblasts and their presence is associated with late stage of senescence. By co‐immunoprecipitation analysis, we show that farnesylated Progerin interacts with human PML2, which accounts for the formation of thread‐like PML NBs. Specifically, human PML2 but not PML1 overexpression in HGPS cells promotes PML thread development and accelerates senescence. Further immunofluorescence microscopy, immuno‐TRAP, and deep sequencing data suggest that these irregular PML NBs might promote senescence by perturbing NB‐associated DNA repair and gene expression in HGPS cells. These data identify irregular structures of PML NBs in senescent HGPS cells and support that the thread‐like PML NBs might be a novel, morphological, and functional biomarker of late senescence.

## Graphical abstract

Progerin–PML2 mediates the formation of thread‐like PML NBs, which perturb NB‐associated DNA repair and gene expression. The thread‐like PML NBs dictate a “0/1 model” of senescence (0 = no senescence; 1 = complete senescence) and thus label late senescence of HGPS cells. Most other senescence markers fit a “0‒1 model”, which requires a threshold value between 0 and 1 to define a senescence stage.

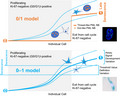

## 1 INTRODUCTION

Promyelocytic leukemia (PML) was originally identified as a fusion oncoprotein with retinoic acid receptor α (RARα). This fusion protein is generated as a result of a chromosomal translocation t(15;17) in acute promyelocytic leukemia (APL) (Lallemand‐Breitenbach & de The, 2010). In mammalian cells, the PML protein forms dot‐like nuclear substructures, known as PML nuclear bodies (PML NBs) that are 0.2–1 μm diameter (Ching, Dellaire, Eskiw, & Bazett‐Jones, 2005). There are six human PML isoforms: They all share the same N‐terminal domain but possess different C termini due to alternative splicing. These different C termini confer different mobilities and diverse functions of PML NBs (Geng et al., 2012; Li, Peng, Wan, Sun, & Tang, 2017; Nisole, Maroui, Mascle, Aubry, & Chelbi‐Alix, 2013). The most abundant isoforms are PML1 and PML2 (Condemine et al., 2006): PML2 is unique to the other five isoforms in that it could localize to the inner nuclear membrane and associate with lipid droplets in some types of cells (Condemine et al., 2006; Jul‐Larsen, Grudic, Bjerkvig, & Boe, 2010; Ohsaki et al., 2016).

Numerous proteins reside in or are temporally recruited to PML NBs (Van Damme, Laukens, Dang, & Van Ostade, 2010). The deposition of various protein partners diversifies PML NB function, enabling this structure to participate in diverse cellular processes, such as cellular senescence, gene transcription, and DNA repair (Hsu & Kao, 2018; Lallemand‐Breitenbach & de The, 2018). For instance, PML NBs are often found co‐localized with or juxtaposed to double‐strand breaks (DSBs) and as such are considered to be DNA damage sensors (Chang et al., 2018). DNA repair factors, such as Rad51, RPA32, NBS1, TopBP1, and BLM, have also been found to deposit in DSB‐associated PML NBs (Chang et al., 2018). Furthermore, several studies have corroborated that PML is involved in homologous recombination (HR) (Boichuk, Hu, Makielski, Pandolfi, & Gjoerup, 2011; Yeung et al., 2012). Increased sister‐chromatid exchange (SCE) was observed in *PML* ^−/−^ cells and *PML^C52A/C65A^* cells with disrupted NBs (Voisset et al., 2018; Zhong et al., 1999). In addition to DNA repair, PML NBs regulate gene transcription either via direct interactions with specific genome loci or by recruiting transcription factors (Aoto, Saitoh, Ichimura, Niwa, & Nakao, 2006; Ching, Ahmed, Boutros, Penn, & Bazett‐Jones, 2013; Ching et al., 2005; Ulbricht et al., 2012; Zhong, Salomoni, & Pandolfi, 2000).

Hutchinson–Gilford progeria syndrome (HGPS) is characterized by premature aging, with an estimated prevalence of 1 in 4–8 million people. HGPS is driven by a de novo mutation in the *LMNA* gene, which yields a farnesylated and truncated prelamin A protein, known as Progerin (Gonzalo, Kreienkamp, & Askjaer, 2017). Progerin accumulation disrupts the nuclear lamina integrity, causing miss‐shaped nuclei, loss of heterochromatin, abnormal epigenetics, and altered gene expression and defective DNA repair (Columbaro et al., 2005; Gonzalo et al., 2017; Hamczyk, del Campo, & Andres, 2018; Liu et al., 2005; Mattioli et al., 2018). Farnesylation is critical for HGPS pathogenesis as nonfarnesylated Progerin protein fails to accelerate aging in mouse models. Nuclear defects in HGPS cells can be largely alleviated by farnesyltransferase inhibitors (FTIs) (Capell et al., 2005; Hamczyk et al., 2018; Toth et al., 2005). However, disruption to other nuclear compartments, such as nuclear bodies, in HGPS is rarely reported (Harhouri et al., 2017). A recent study identified disordered structures of PML NB in late passage of cultured HGPS cells (Harhouri et al., 2017); this study, however, did not clarify their function or effects on cellular processes.

In this study, we aimed to study the roles of PML NBs in HGPS pathogenesis. We show that the presence of aberrantly reorganized thread‐like PML NB structures in HGPS cells is closely associated with senescence. Mechanistically, we demonstrate that farnesylated Progerin specifically associates with PML2, mediating the formation of thread‐like PML NBs. Human PML2 overexpression promotes the development of PML threads and accelerates senescence. We uncover that irregular PML NBs perturb NB‐associated DNA repair and gene transcription. These data thus reveal a marker for late senescence and shed light on the mechanisms of defective DNA repair and deregulated gene expression in HGPS cells.

## 2 RESULTS

### 2.1 Thread‐like PML NBs are associated with late senescence in HGPS cells

**FIGURE 1:**
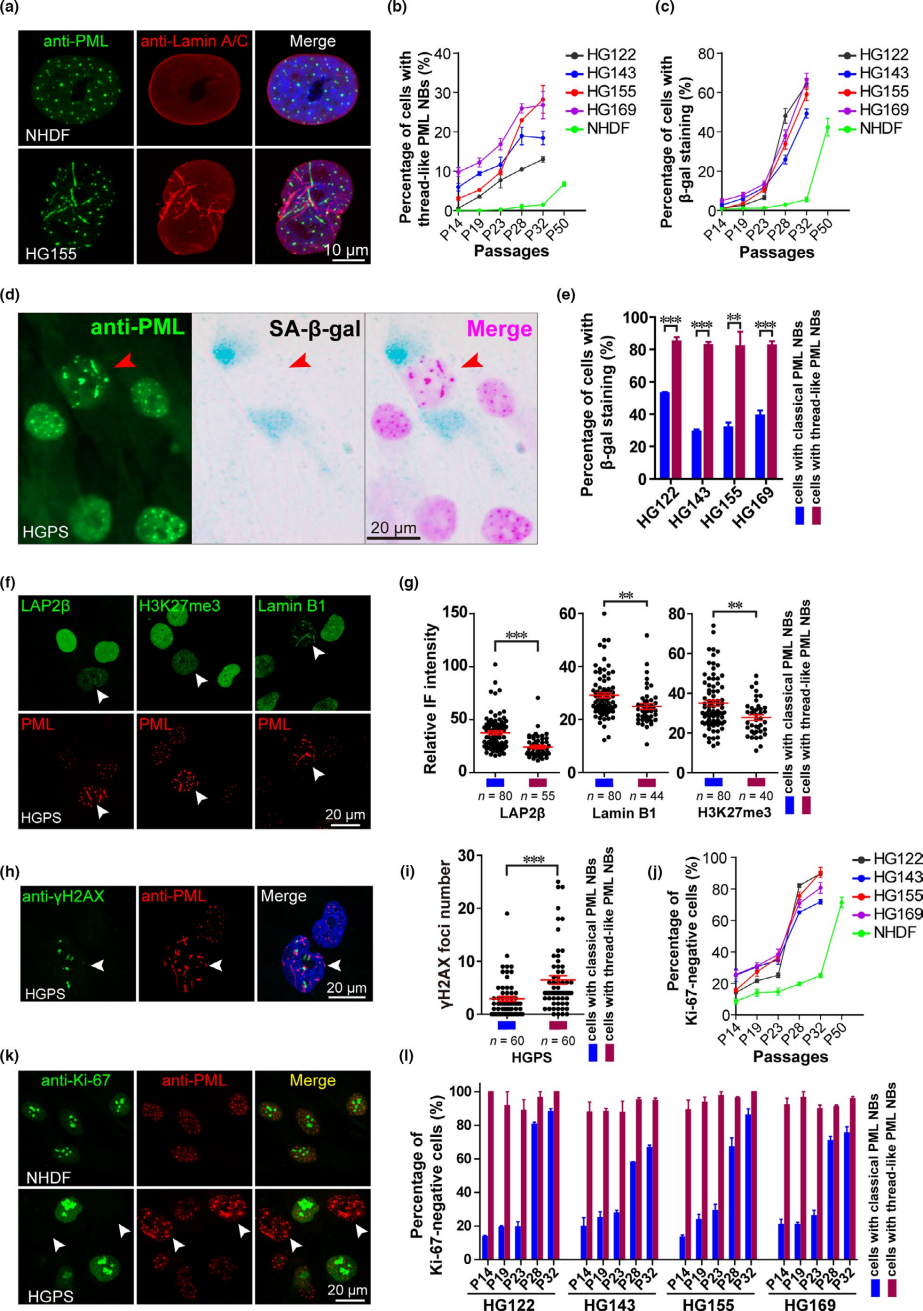
Thread‐like PML NBs are associated with senescence. (a) Normal human dermal fibroblasts (NHDFs) and HGADFN155 (HG155) cells were stained with anti‐PML and Lamin A/C antibodies. The nuclei were counterstained with DAPI. The representative images show thread‐like PML NBs. Scale bar, 10 μm. (b, c) The percentage of cells with thread‐like PML NBs (b) or β‐gal‐positive staining (c) was determined in NHDF and four HGPS cell lines across different passages. (d) SA‐β‐gal staining and PML immunolabeling were performed in HGPS cells. The arrows indicate cells with thread‐like PML NBs. Scale bar, 20 μm. (e) The percentage of cells with β‐gal staining was analyzed in four HGPS cell lines at passage 28 with normal or thread‐like PML NBs. **p < .01, ***p < .001. (f) PML, LAP2β, H3K27me3, and Lamin B1 immunolabeling in HGPS cells at passage 28. The arrows indicate cells with thread‐like PML NBs. Scale bar, 20 μm. (g) Statistical analysis of the immunofluorescent (IF) signal intensity for LAP2β, H3K27me3, and Lamin B1 staining in HGPS cells with normal or thread‐like PML NBs. **p < .01, ***p < .001. (h) γH2AX and PML were stained in HGPS cells at passage 28. Scale bar, 20 μm. (i) The γH2AX foci number in HGPS cells with normal or thread‐like PML NBs. ***p < .001. (j) The percentage of cells with Ki‐67‐negative staining was determined in NHDF and four HGPS cell lines across different passages. (k) NHDF and HGPS cells were stained with anti‐PML and Ki‐67 antibodies. HGPS cells with thread‐like PML NBs are indicated by arrows. Scale bar, 20 μm. (l) The percentage of Ki‐67‐negative cells with normal or thread‐like PML NBs was determined in four HGPS cell lines across different passages

In normal human cells, PML NBs are normally present as dot‐like structures in the nucleus (Lallemand‐Breitenbach & de The, 2010). Interestingly, we found that PML NBs were aberrantly organized into thread‐like structures in a significant proportion of HGPS cells at late passage, ranging from ~13% to ~28% in four cell lines derived from individual HGPS patients—HG122, HG143, HG155, and HG169 (Figure 1a,b, and Figure S1a,b). Moreover, the percentage of cells with thread‐like PML NBs progressively increased with subsequent cell passaging (Figure 1b).

Elevated senescence‐associated β‐galactosidase (SA‐β‐gal) activity, persistent γH2AX foci, decreased levels of LAP2β, H3K27me3, and Lamin B1 collectively mark the deficiency and senescence of HGPS cells (Kubben et al., 2016; Liu et al., 2005, 2011). Here, we found >80% HGPS cells with thread‐like PML NBs exhibited positive SA‐β‐gal staining at P28 (Figure 1c–e), indicating that these cells were undergoing senescence. The levels of LAP2β, H3K27me3, and Lamin B1 in HGPS cells with thread‐like PML NB were, respectively, 35.9%, 20.8%, and 14.3% lower than that in HGPS cells with classical dot‐like PML NBs (Figure 1f,g). In addition, HGPS cells with thread‐like PML NBs showed a 2.2‐fold higher number of γH2AX foci compared to those with classical PML NBs (Figure 1h,i).

Senescent cells permanently exit from the cell cycle and can be identified by the absence of Ki‐67 staining (Gorgoulis et al., 2019). Significantly, the percentage of Ki‐67‐negative cells increased sharply in HGPS cells with passaging (Figure 1j) and that the Ki‐67 signal was rarely seen in cells with thread‐like PML NBs (Figure 1k,l).

Taken together, thread‐like PML NBs could serve as a morphological marker and are correlated with a severe deficiency of HGPS cells. We believe that it is reasonable to define HGPS cells with thread‐like PML NBs, most of which are SA‐β‐gal‐positive but Ki‐67‐negative, express relatively low levels of LAP2β, H3K27me3, and Lamin B1, and contain a higher number of persistent γH2AX foci, as a state of late senescence. Interestingly, such thread‐like PML NBs were also observed in ~7% of late‐passage normal human dermal fibroblasts (NHDFs) (Figure 1b and Figure S1c). Further analyses revealed that over 90% of these NHDF cells with thread‐like PML NBs are Ki‐67‐negative and 83.5% are β‐gal‐positive (Figure S1d), suggesting that the thread‐like PML NBs might also dictate a late stage of replicative senescence.

### 2.2 Thread‐like PML NBs compromise genomic stability

**FIGURE 2:**
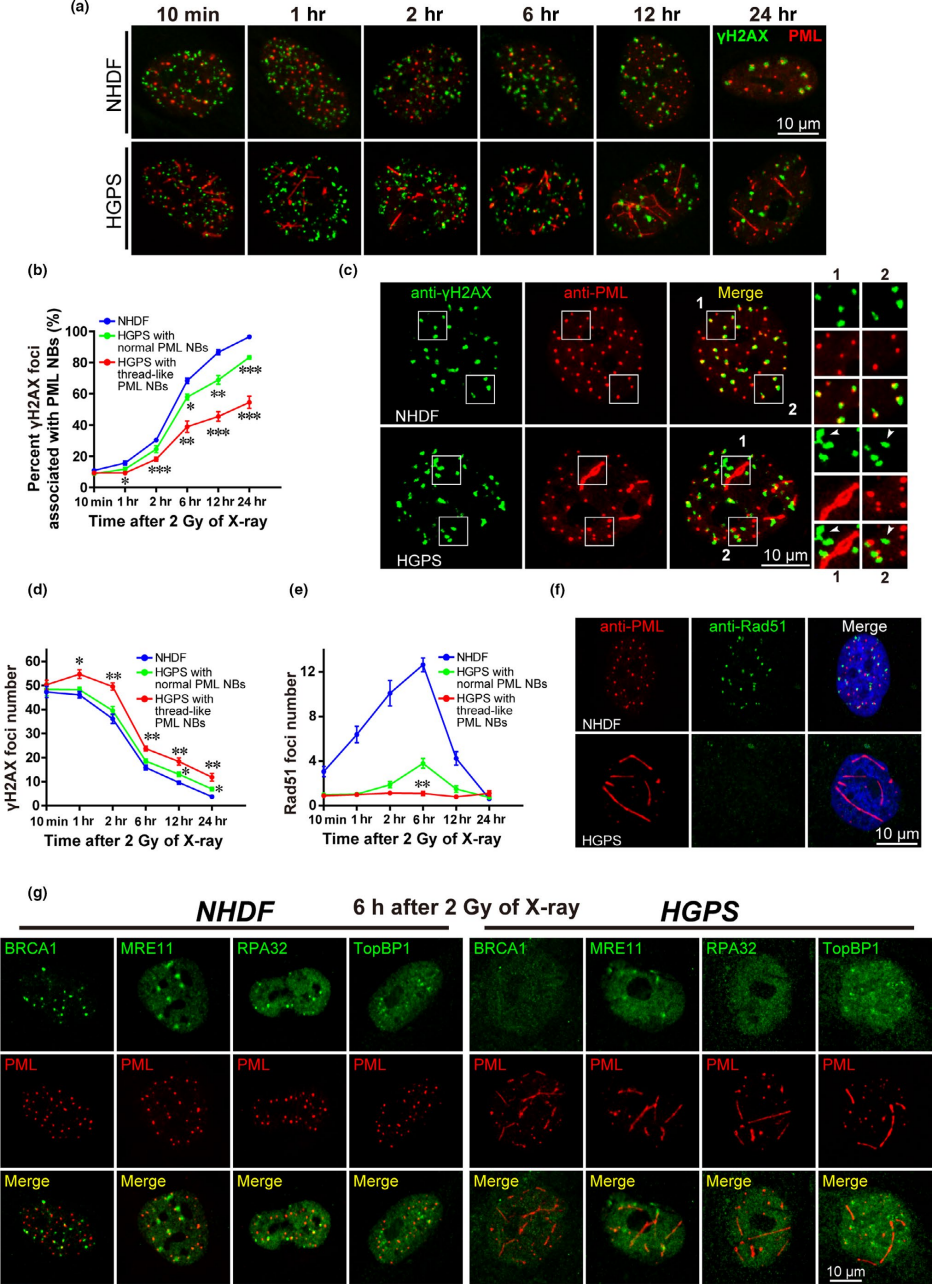
Thread‐like PML NBs compromise DNA repair. (a) Immunofluorescent (IF) γH2AX and PML staining in NHDF and HGPS cells (at passage 23) at the indicated time points after exposure to 2 Gy of X‐ray irradiation. Scale bar, 10 μm. (b) The percentage of γH2AX foci associated with PML NBs in NHDF and HGPS cells, with normal or thread‐like PML NBs, at the indicated time points after exposure to 2 Gy X‐ray irradiation. *p < .05, **p < .01, ***p < .001, compared with the NHDF group. (c) γH2AX and PML IF staining in NHDF and HGPS cells 12 hr after exposure to 2 Gy X‐ray irradiation. In the magnified images (labeled 1 and 2) show the association between γH2AX foci and PML NBs. The arrows indicate PML NBs that are not associated with γH2AX foci. Scale bar, 10 μm. (d) A time‐course analysis of γH2AX foci formation and persistence in NHDF and HGPS cells after exposure to 2 Gy X‐ray irradiation. *p < .05, **p < .01, compared with NHDF group. (e) A time‐course analysis of Rad51 foci formation in NHDF and HGPS cells (at passage 23) after 2 Gy of X‐ray irradiation. **p < .01, compared to the HGPS with normal PML NBs at 6 hr after irradiation. (f) Rad51 and PML IF staining in NHDF and HGPS cells at 6 hr after exposure to 2 Gy X‐ray irradiation. Scale bar, 10 μm. (g) PML, BRCA1, NRE11, RPA32, and TopBP1 IF staining in NHDF and HGPS cells (at passage 23) at 6 hr after exposure to 2 Gy X‐ray irradiation. Scale bar, 10 μm

PML NBs are implicated in DNA repair processes (Chang et al., 2018). We reasoned that thread‐like PML NBs might not only serve as a phenotypic marker, but also functionally compromise genomic stability to accelerate senescence in HGPS cells. To test this hypothesis, we exposed NHDF and HGPS cells to 2 Gy of X‐ray irradiation and monitored the DNA repair process in terms of γH2AX foci formation and persistence. As shown, the number of PML NB‐associated γH2AX foci was notably less in HGPS cells with thread‐like PML NBs than in HGPS cells with normal PML NBs (Figure 2a). In NHDF cells, we found that 12 hr after irradiation, >86% of γH2AX foci were co‐localized with or juxtaposed to PML NBs, whereas in HGPS cells with thread‐like NBs, no more than 50% of γH2AX foci were associated with PML NBs (Figure 2b,c). Notably, in HGPS cells with thread‐like PML NBs, while thread‐like PML NBs were rarely co‐localized with γH2AX foci, dot‐like PML NBs were still co‐localized with a fraction of γH2AX foci (Figure 2c).

When looking at γH2AX foci formation and persistence after X‐ray irradiation over time, we noticed that the number of induced DNA lesions was comparable between NHDF and HGPS cells at 10 min after irradiation. However, we found much more unrepaired foci remained in HGPS cells with thread‐like PML NBs at 1 hr after irradiation or later at the same time point. These data suggest that DNA repair is defective in cells with thread‐like PML NBs (Figure 2d).

Given the important roles of PML NBs in HR‐mediated DSB repair (Boichuk et al., 2011; Yeung et al., 2012), we next examined the effect of thread‐like PML NBs on the expression of Rad51—a recombinase that is critical for HR—in irradiated cells. We rarely observed Rad51 foci in HGPS cells with thread‐like PML NBs, whereas we detected a certain amount of Rad51 foci in HGPS cells with classical PML NBs (Figure 2e,f). These data suggest that HR is severely compromised in cells with thread‐like NBs. We also noted that the associations of BRCA1, MRE11, RPA32, and TopBP1 with PML NBs were also perturbed in HGPS cells with thread‐like NBs (Figure 2g). These findings support that normal PML NBs are involved in DNA repair and that thread‐like PML NBs are indicators of defective DNA repair in HGPS cells. These thread‐like structures seem to disturb the normal association of PML NBs and DNA lesions and compromise genomic stability.

### 2.3 Progerin hijacks PML2 to mediate thread‐like PML NB formation

**FIGURE 3:**
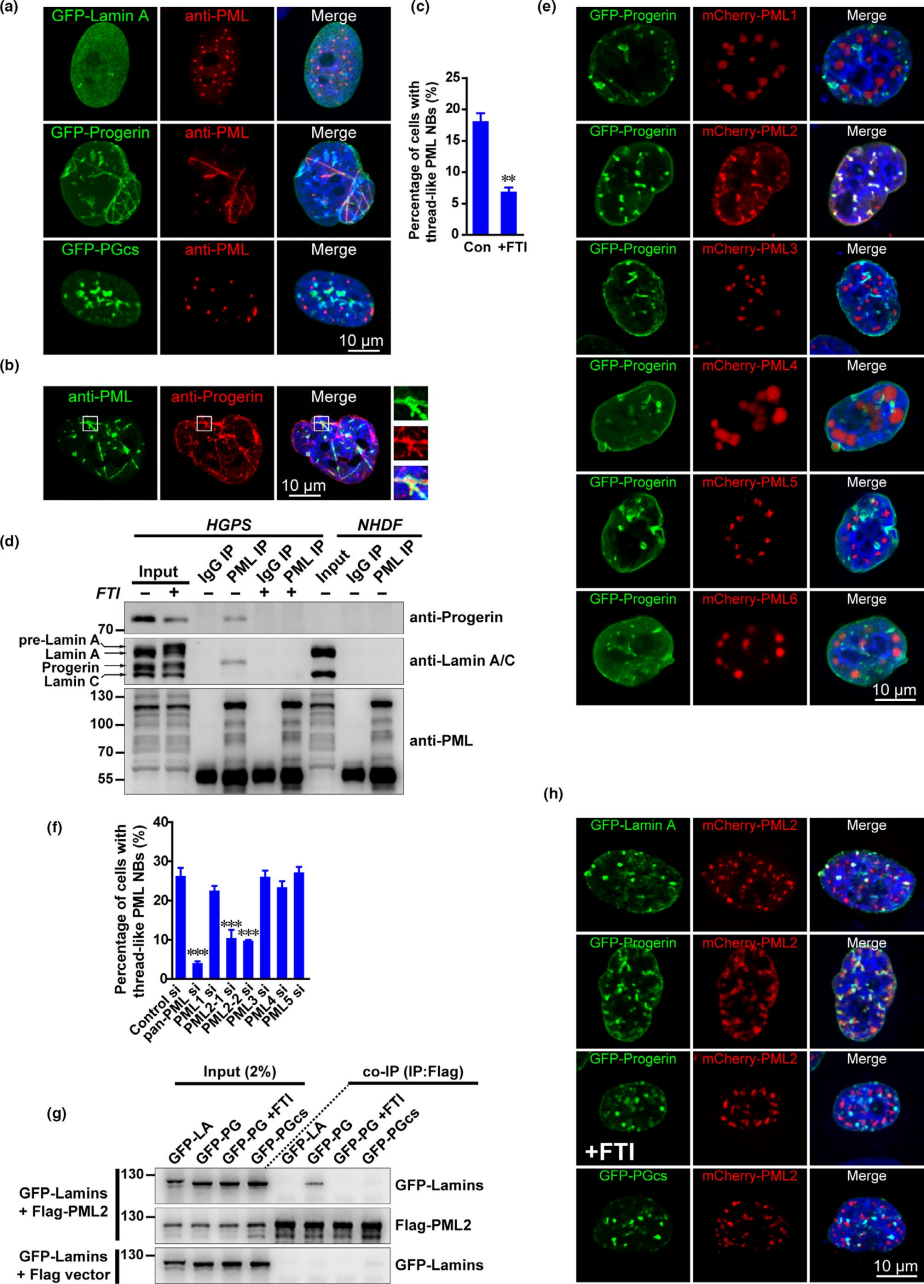
Progerin interacts with PML2 to induce thread‐like PML NBs. (a) Immunofluorescent (IF) PML staining in NHDF cells expressing GFP–Lamin A, GFP–Progerin, and an unfarnesylated mutant, GFP‐PGcs. Scale bar, 10 μm. (b) PML and Progerin IF staining in HGPS cells. Co‐localization is shown in the magnified images (right panels). Scale bar, 10 μm. (c) HGPS cells at passage 26 were treated with the farnesylation inhibitor lonafarnib (2 μM) combined with zoledronate (1 μM) (referred to FTI) for 3 days, and the percentage of HGPS cells with thread‐like PML NBs was determined. **p < .01. (d) NHDF and HGPS cells (at passage 28) were treated with or without the FTI for 3 days and then lysed for immunoprecipitation (IP) using PML or IgG control antibodies. Progerin, Lamin A/C, and PML were analyzed by western blotting. (e) GFP–Progerin was co‐transfected with mCherry‐PML isoforms 1–6 in HeLa cells for 18 hr, cells were fixed, and the nuclei were counterstained with DAPI. Scale bar, 10 μm. (f) HGPS cells at passage 28 were transfected with control siRNA or siRNAs specifically targeted to pan‐PML or individual PML isoforms for 72 hr, and then fixed for IF staining with a PML antibody. The percentage of cells with thread‐like PML NBs was determined. ***p < .001, compared with the control group. (g) FLAG‐PML2 or an empty FLAG vector was co‐transfected with GFP–Lamin A (LA), GFP–Progerin (PG), or an unfarnesylated mutant, GFP‐PGcs, in HEK293T cells for 18 hr. Lonafarnib (4 μM) combined with zoledronate (2 μM) (FTI) was used to inhibit farnesylation. Cells were lysed for IP with an anti‐FLAG antibody. (h) mCherry‐PML2 was co‐transfected with GFP–Lamin A, GFP–Progerin, or GFP‐PGcs in HeLa cells for 18 hr. Lonafarnib (4 μM) combined with zoledronate (2 μM) (FTI) was used to inhibit farnesylation. The cells were fixed, and the nuclei were counterstained with DAPI. Scale bar, 10 μm

We next wanted to determine the composition of thread‐like PML NBs and how they form. Here, we found that exogenous Progerin but not Lamin A or nonfarnesylated Progerin (PGcs) expression induced the formation of thread‐like PML NBs in NHDF cells (Figure 3a). Furthermore, Progerin co‐localized with these thread‐like PML NBs (Figure 3a). We also observed prominent co‐localization between endogenous Progerin and thread‐like PML NBs in a fraction of HGPS cells (Figure 3b), suggesting that Progerin could associate with thread‐like PML NBs directly. Interestingly, treatment with the inhibitor FTI, which inhibits Progerin farnesylation, significantly reduced the percentage of thread‐like PML NBs in HGPS cells (Figure 3c). These results suggest a direct role for farnesylated Progerin in reshaping PML NB morphology.

We next performed co‐immunoprecipitation (CoIP) experiments to show that Progerin is present in anti‐PML immunoprecipitates from HGPS cells (Figure 3d). Consistent with our earlier findings, FTI treatment abolished this PML–Progerin association. By contrast, interaction between PML and Lamin A/C was merely detected in HGPS and NHDF cells (Figure 3d).

Human PML has six isoforms as a result of alternative splicing, and data suggest that these isoforms have distinct characteristics and functions (Nisole et al., 2013). Our CoIP assays confirmed that Progerin specifically interacted with human PML2 (Figure S2a). Immunofluorescence (IF) staining also showed that only PML2 co‐localized with Progerin in HeLa cells (Figure 3e). To investigate the contributing role of the different PML isoforms in thread‐like PML NB formation, we knocked down each individual PML isoform (1–5) by RNA interference and monitored mRNA level (Figure S2b). Only PML2 knockdown significantly reduced the percentage of cells with thread‐like PML NBs (Figure 3f).

Our final CoIP and IF analyses showed that Progerin farnesylation was necessary for the PML2 and Progerin interaction, as FTI treatment abolished their biochemical association and cellular co‐localization (Figure 3g,h). In addition, exogenous Progerin expression in HeLa cells resulted in PML2 recruitment to the nuclear membrane and subsequent reforming of the PML NBs into thread‐like structures (Figure 3h). These findings imply that PML2 specifically promotes the formation of thread‐like PML NBs through farnesylated Progerin.

### 2.4 PML2 overexpression accelerates senescence in HGPS cells

**FIGURE 4:**
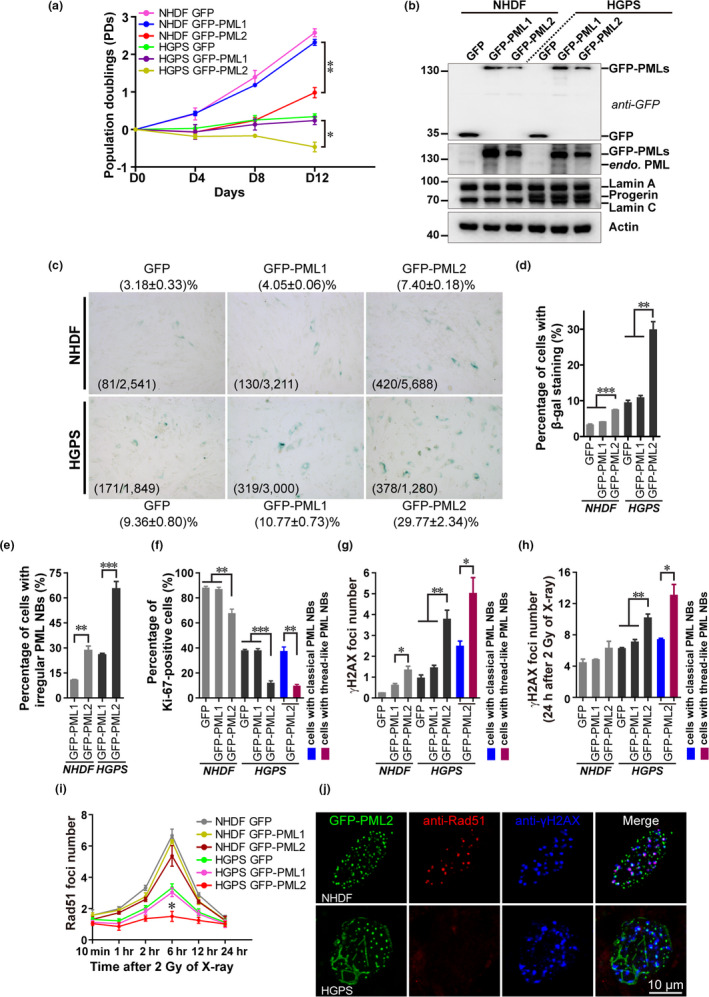
PML2 promotes irregular PML NB formation and accelerates cell senescence. (a) NHDF and HGPS cells at passage 19 were infected with a lentivirus expressing GFP, GFP‐PML1, or GFP‐PML2. After selection with puromycin, the cells were passaged every 4 days and population doublings (PDs) were analyzed. The PDs on day 12 after transduction were compared. *p < .05, **p < .01. (b) Western blot analysis of NHDF and HGPS cells expressing GFP, GFP‐PML1, or GFP‐PML2. (c) NHDF and HGPS cells expressing GFP, GFP‐PML1, or GFP‐PML2 were fixed for SA‐β‐gal staining. The number of β‐gal‐positive cells over the total number of cells is shown. (d) The percentage of NHDF and HGPS cells with positive β‐gal staining was determined. **p < .01, ***p < .001. (e) NHDF and HGPS cells expressing GFP‐PML1 or GFP‐PML2 were fixed, and the percentage of cells with irregular PML NBs was determined. **p < .01, ***p < .001. (f) The percentage of Ki‐67‐positive NHDF and HGPS cells expressing GFP, GFP‐PML1, or GFP‐PML2, and Ki‐67‐positive GFP‐PML2 HGPS cells with normal or thread‐like PML NBs was determined. **p < .01, ***p < .001. (g) The number of γH2AX foci in NHDF and HGPS cells expressing GFP, GFP‐PML1, or GFP‐PML2, and the γH2AX foci number in HGPS cells expressing GFP‐PML2 with normal or thread‐like PML NBs was determined. *p < .05, **p < .01. (h) NHDF and HGPS cells expressing GFP, GFP‐PML1, or GFP‐PML2 were exposed to 2 Gy X‐ray irradiation. After 24 hr, the γH2AX foci number was determined in cells with either normal or thread‐like PML NBs. *p < .05, **p < .01. (i) Time‐course analysis of Rad51 foci formation in NHDF and HGPS cells expressing GFP, GFP‐PML1, or GFP‐PML2 after exposure to 2 Gy X‐ray irradiation. *p < .05, compared with the HGPS GFP‐PML1 group. (j) γH2AX and Rad51 IF staining in NHDF and HGPS cells expressing GFP‐PML2 at 6 hr after exposure to 2 Gy X‐ray irradiation. Scale bar, 10 μm

As PML2 contributes to the formation of thread‐like PML NBs, which correlates with senescence as demonstrated by our earlier findings, we next analyzed the roles of PML2 in HGPS cell senescence. The PML isoforms PML1 and PML2 have a comparable molecular weight and are the most abundant in human cells (Condemine et al., 2006). As no effect of PML1 on thread‐like PML NB formation was observed, we used this isoform as a control in our assays going forward. Cell proliferation assays revealed that the exogenous expression of GFP‐PML2, but not GFP‐PML1, notably inhibited NHDF and HGPS cell growth (Figure 4a,b). Although both GFP‐PML2 and GFP‐PML1 had negligible effects on NHDF cell senescence, SA‐β‐gal staining showed that GFP‐PML2 accelerated senescence in HGPS cells as determined by 2.76‐fold higher percent of SA‐β‐gal‐positive staining compared with GFP‐PML1 (Figure 4c,d).

We then characterized the association between PML NB morphology and DNA repair processes in PML overexpressing cells. In 90% of NHDF cells, GFP‐PML1 formed NBs with a classical dot‐like morphology. By contrast, 28% of NHDF cells transfected with GFP‐PML2 exhibited abnormal PML NBs. More noticeably,> 65% HGPS cells expressing GFP‐PML2 developed abnormal, thread‐like PML NBs (Figure 4e). Moreover, the percentage of Ki‐67‐positive cells decreased, while the number of γH2AX foci increased in NHDF and HGPS cells expressing GFP‐PML2 compared with GFP‐PML1, especially in cells with thread‐like NBs (Figure 4f,g, and Figure S3a,b). After X‐ray irradiation, 1.44‐fold higher number of γH2AX foci remained in HGPS cells expressing GFP‐PML2 compared with that of GFP‐PML1, again suggesting defective DNA repair (Figure 4h, and Figure S3c,d). Moreover, Rad51 recruitment to DNA lesions was completely abolished in HGPS cells overexpressing GFP‐PML2 but maintained in GFP‐PML1 overexpressing cells (Figure 4i,j). Together, these results suggest that accumulated PML2 protein promotes HGPS cell senescence, accompanied by abnormal, thread‐like PML NBs and deficient DNA repair.

### 2.5 PML2‐dictated gene signature in HGPS cells

**FIGURE 5:**
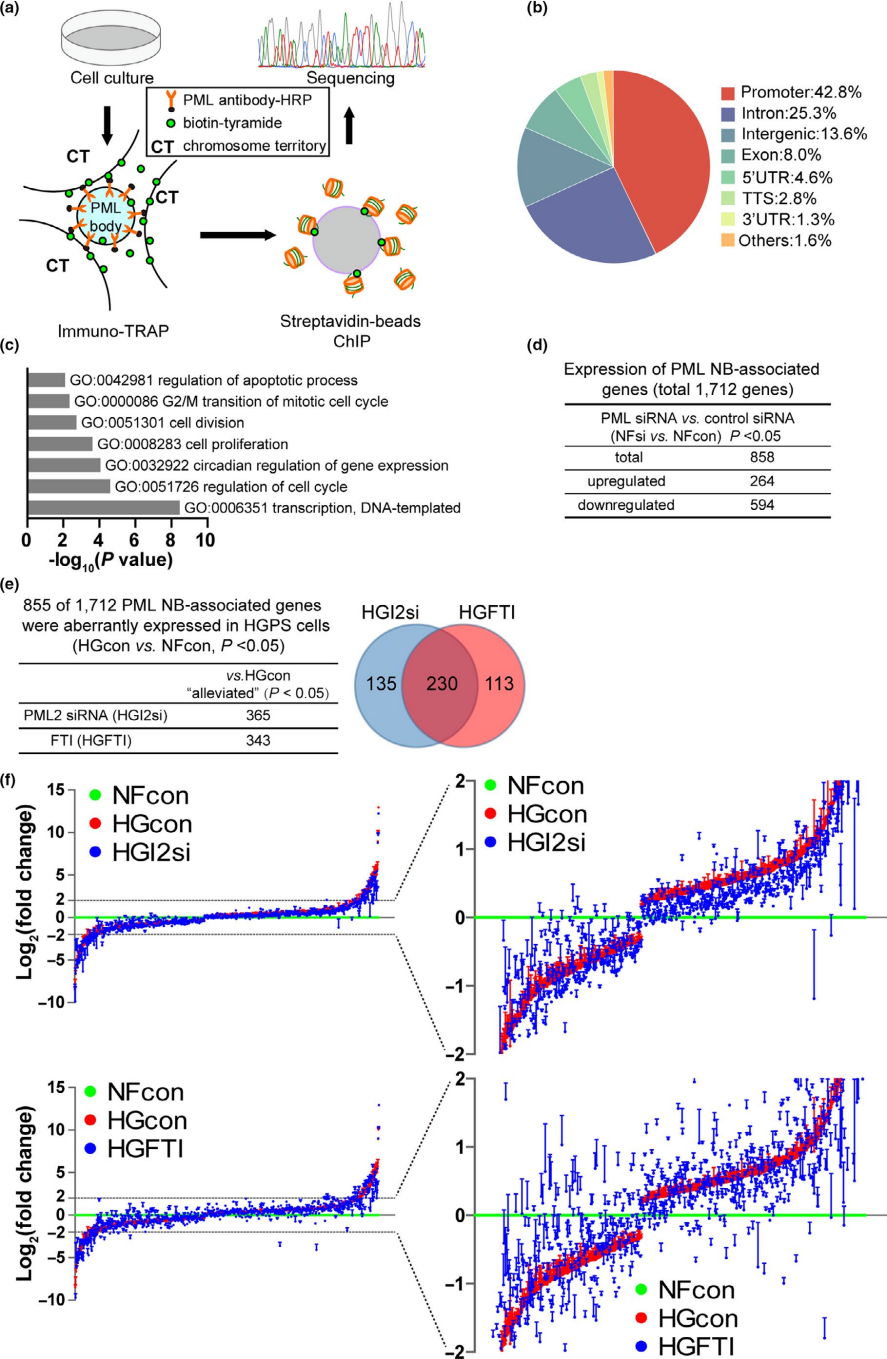
Progerin–PML2 deregulates NB‐associated gene expression in HGPS cells. (a) Scheme of the immuno‐TRAP experiment to systematically analyze PML NB‐associated genes. (b) Pie chart representation of the genomic location distribution of 1,972 differential peaks identified from the ChIP‐seq data. (c) GO term enrichment analysis of 1,712 PML NB‐associated genes. (d) Analysis of the differential gene expression profile between NHDF control siRNA (NFcon) and NHDF PML siRNA (NFsi) based on RNA‐seq data: 858/1,712 PML NB‐associated differentially expressed genes were identified. A total of 264 genes were upregulated and 594 genes were downregulated in the NFsi group compared with the NFcon group. (e) Analysis of the differential gene expression profiles between NHDF (NFcon) and HGPS (HGcon) cells (at passage 28) based on RNA‐seq data: 855/1,712 PML NB‐associated genes were aberrantly expressed in HGPS cells compared with NHDF cells. Among these 855 genes, the expressions of 365 and 343 genes were restored by PML2 siRNA (HGI2si) and FTI (HGFTI) treatment (at passage 28), respectively. The Venn diagram shows the degree of overlap in the significantly restored genes between the two groups. (f) The expression of deregulated PML NB‐associated genes was normalized to the NFcon group, and the Log_2_(fold change) analysis of gene expression in HGcon, HGI2si, and HGFTI groups is presented

PML NBs associate with gene loci to regulate transcription (Aoto et al., 2006; Ching et al., 2005, 2013; Ulbricht et al., 2012). To delineate the functional relevance of PML2 in HGPS cell senescence, we investigated the PML‐dictated gene signature in HGPS cells. We first used an immuno‐TRAP method (Ching et al., 2013) combined with chromatin immunoprecipitation (ChIP) and deep sequencing to identify PML NB‐associated gene loci in NHDF cells (Figure 5a). We confirmed successful immuno‐TRAP by the co‐localization of PML NBs with biotin‐tyramide signals (Figure S4a). We detected 1,972 different peaks by ChIP‐seq; 1,712 of these peaks were identified by annotation and designated as PML‐associated gene set (Files S1 and S2). Notably, 844/1,972 peaks (42.8%) were located in promoter regions of genes (Figure 5b, and Figure S4b), such as the gene *DDIT4*, which was recently shown to be associated with PML NBs (Salsman et al., 2017). Gene Ontology (GO) analysis revealed that the PML NB‐associated genes were enriched in pathways regulating gene transcription, cell cycle, circadian rhythms, and apoptosis (Figure 5c, and File S3).

To confirm whether these genes are indeed transcriptionally modulated by PML, we transfected NHDF cells with a siRNA targeting pan‐PML and extracted mRNA for sequencing. From the 1,712 identified PML NB‐associated genes, we found that 858 genes were differentially expressed in the PML siRNA‐treated NHDF cells compared with the control siRNA‐treated cells (*p* < .05): 264 genes were significantly upregulated and 594 were significantly downregulated (Figure 5d, and Files S4 and S5). These results suggest that PML regulates the transcription of a specific set of genes to constitute a PML‐gene signature.

We next examined whether our identified PML‐gene signature was deregulated in HGPS cells. By comparing the transcriptomes, we found that 855 of 1,712 PML signature genes were aberrantly expressed in HGPS cells compared with NHDF cells (Figure 5e, and File S6). As both suppression of PML2 by siRNA and Progerin farnesylation by FTI treatment could alleviate the irregular PML NB structures in HGPS cells (Figure 3c,f), we explored the effects of these treatments on PML signature gene expression. Of the 855 aberrantly expressed genes, 365 were restored in PML2 siRNA‐treated HGPS cells and 343 were restored in FTI‐treated cells (*p* < .05) (Figure 5e,f). Of note, 230 of these genes overlapped between the PML2 siRNA‐treated and FTI‐treated groups (Figure 5e, and File S6). Collectively, these data suggest that an aberrant Progerin–PML2 association disrupts the PML NB‐gene signature in HGPS cells.

## 3 DISCUSSION

A challenge in research on aging is the lack of a single marker or gold standard with absolute specificity for senescence. Currently, senescence must be defined by a combination of multiple factors and/or features (Gorgoulis et al., 2019). Here, we identified thread‐like PML NBs as a more definitive morphological and functional marker of senescence in HGPS cells. We found that most HGPS cells with thread‐like PML NBs are SA‐β‐gal‐positive but Ki‐67‐negative, express relatively lower levels of LAP2β, H3K27me3, and Lamin B1, and contain more persistent γH2AX foci. We thus consider that thread‐like PML NBs most likely mark a stage of late senescence in HGPS cells.

SA‐β‐gal is widely used as senescence marker, but it is neither required nor a determinant of a senescent phenotype (Gorgoulis et al., 2019; Hernandez‐Segura, Nehme, & Demaria, 2018), not to mention that the intensity of SA‐β‐gal signal varies with developing time. Reduced levels of LAP2β, H3K27me3, and Lamin B1 are also prominent in HGPS cells. Notably, all these markers are progressively increased or downregulated with cell passaging. We summarize these parameters to a “0‒1 model,” whereby a threshold between 0 and 1 has to be included to define a senescence stage (0 = no senescence; 1 = complete senescence). Thus, “0‒1 model”‐defined senescence is contextual. Here, we identified thread‐like PML NBs as a potential senescence marker at least in HGPS cells. The presence of these threads fits a “0/1 model,” that is, a yes or no model, that we consider, is more straightforward and definitive.

Although thread‐like PML NBs were recently described in HGPS (Harhouri et al., 2017) and were thought to sequester Progerin to promote degradation, we found no changes in Progerin levels in PML shRNA‐treated HGPS cells (data not shown). The thread‐like PML NB structures not only serve as a phenotypic marker, but also impede DNA repair in HGPS cells. Although the precise roles of PML NBs in DNA repair remain unclear, a current hypothesis is that PML NBs might function as a platform to coordinate DNA repair factors (Chang et al., 2018). Here, we found that the DNA repair factors Rad51, BRCA1, MRE11, RPA32, and TopBP1 are excluded from thread‐like PML NBs. We thus propose that thread‐like PML NBs are functional and might compromise DNA repair by disorganizing DNA repair partners.

Owing to their insolubility, PMLs immunoprecipitated using the traditional IP method are predominantly not localized to NBs (Delbarre et al., 2017). We applied a modified immuno‐TRAP technique to overcome the issue and systematically analyze the PML NB‐associated genes or the PML NB‐dictated gene signature (Ching et al., 2013). Our findings demonstrate that PML NBs could associate directly with gene loci to regulate gene expression. The identification of PML NB‐gene signature may help clarify diversified roles of PML NBs in various biological activities. In addition, we found that almost half of the PML NB‐dictated genes were deregulated in HGPS cells, and >40% of them could be restored by PML2 siRNA or FTI treatment. The rescue effects elicited by human PML2 siRNA and FTI treatment on PML NB‐gene signature showed notable overlap (> 63%), and many of them are associated with senescence (Figure 5e,f and Files S6 and S7). Given that Progerin, PML2, and the Progerin–PML2 association seem equally important for regulating the PML‐gene signature, these data further support thread‐like PML NBs as a functional biomarker of senescence in HGPS.

PML NBs concentrate lots of partner proteins to regulate versatile cellular processes (Lallemand‐Breitenbach & de The, 2018; Van Damme et al., 2010). Thus, in addition to DNA repair and gene expression, thread‐like PML NBs may also perturb other PML NB‐associated cellular processes, possibly contributing to HGPS deficiency. ATRX and DAXX are residents of PML NBs, which have been identified as H3.3 histone chaperones (Lewis, Elsaesser, Noh, Stadler, & Allis, 2010). Recent studies reveal that the PML‐ATRX/DAXX complex associates with chromatin and regulates H3.3 deposition to modulate epigenetics, heterochromatin domain, and DNA replication (Delbarre et al., 2017; Lallemand‐Breitenbach & de The, 2018; Shastrula et al., 2019). Since the loss of heterochromatin, disorganized epigenetics, and abnormal DNA replication are well‐known features of HGPS, we reason that thread‐like PML NBs may perturb the regular function of PML‐ATRX/DAXX complex, thereby contributing to HGPS deficiency.

While the mechanism is still unclear, we show that of the six PML isoforms, only PML2 contributes to thread‐like NB formation. Interestingly, there is no PML2 ortholog in mice (Condemine et al., 2006), and consistently, we rarely observed thread‐like PML NBs in mouse embryonic fibroblasts (MEFs) isolated from a progeroid mouse model (*Zmpste24* ^−/−^ mice; data not shown) (Ao et al., 2019). Exogenous expression of human *PML2* but not *PML1* did, however, induce thread‐like NBs and promoted senescence in *Zmpste24* ^−/−^ MEFs (data not shown). These data further support that PML2 is critical for the formation of thread‐like NBs and accelerated senescence in HGPS. Of note, this finding also points out a limitation of using mouse models to fully unravel the molecular mechanisms of HGPS.

Collectively, we reveal that Progerin sequesters PML2, triggering the formation of thread‐like PML NBs, which identify HGPS cells in late senescence. Thread‐like PML NBs perturb DNA repair, alter the unique PML NB‐associated gene signature, and promote senescence. As thread‐like PML NBs were also observed in late‐passage normal human cells, it might dictate a late stage of replicative senescence.

## 4 EXPERIMENTAL PROCEDURES

### 4.1 Cell culture and treatment

HGPS dermal fibroblasts, including HGADFN122 (HG122), HGADFN143 (HG143), HGADFN155 (HG155), and HGADFN169 (HG169), were described previously (Liu et al., 2005). Primary normal human dermal fibroblasts (NHDFs) were isolated from a healthy female donor (aged 24 years). All cells were maintained in DMEM supplemented with 15% FBS, 0.1 mM NEAA, 2 mM GlutaMAX^®^ and 50 U/ml penicillin, 50 μg/ml streptomycin, at 37°C in a humidified incubator with 5% CO_2_. The cells were passaged every 3 days. Population doubling (PD) was determined by the formula log_2_(*A*/*B*), where *A* is the harvested cell number and *B* is the initially seeded cell number (5 × 10^5^). To inhibit farnesylation in HGPS cells, the farnesyltransferase inhibitor lonafarnib (S2797, Selleck) (2 μM) combined with zoledronate (S1314, Selleck) (1 μM) (Capell et al., 2005; Toth et al., 2005; Varela et al., 2008) was used and referred to as FTI in this study. For the treatment of FTI in HeLa cells, 4 μM of lonafarnib combined with 2 μM of zoledronate was used.

### 4.2 Transfection and virus infection

Details are described in Supplementary Material[Link].

### 4.3 X‐ray irradiation

Details are described in Supplementary Material[Link].

### 4.4 Immunofluorescence microscopy

Details are described in Supplementary Material[Link].

### 4.5 Immunoprecipitation and Western blotting

Details are described in Supplementary Material[Link].

### 4.6 Senescence‐associated β‐galactosidase (SA‐β‐gal) staining

Details are described in Supplementary Material[Link].

### 4.7 RNA extraction and qRT–PCR

Details are described in Supplementary Material[Link].

### 4.8 Immuno‐TRAP and ChIP

The immuno‐TRAP technique was performed as previously described (Ching et al., 2013), with modifications. In brief, 1 × 10^7^ cells cultured in 10‐cm dishes were fixed with 2% PFA in PBS for 10 min at room temperature (RT) and then quenched with 0.1 M glycine in PBS for 10 min. After permeabilization with 0.5% Triton X‐100 in PBS, endogenous peroxidase activity was quenched by 0.5% H_2_O_2_ in PBS for 10 min at RT. Then, 3% BSA prepared in TST buffer (100 mM Tris‐HCl pH 7.5, 150 mM NaCl, 0.05% Tween‐20) was used to block cells for 1 hr at RT. PML antibodies conjugated with horse‐radish peroxidase (PG‐M3, sc‐966 HRP, Santa Cruz) were diluted at 1:300 in chilled 3% BSA in TST and incubated at 4°C overnight. The cells were rinsed with borate buffer solution (BBS; 100 mM boric acid, 25 mM sodium tetraborate, 75 mM NaCl, pH 8.5) and incubated with freshly prepared reaction buffer (3.3 μg/ml biotinylated tyramide and 0.003% H_2_O_2_ in BBS) for 3 min at 26°C. The enzymatic reaction was stopped by incubating with 0.5% H_2_O_2_ at RT for 10 min. To qualify the reaction, streptavidin conjugated with Alexa Flour^®^ 568 (S11226, Life Technologies) was used to detect biotin‐labeled targets by immunofluorescence staining.

For ChIP assay, the cells were scraped off in 1× PBS containing 0.5% BSA and collected into a 50‐ml Eppendorf tube. The cells were pelleted at 750 *g* for 8 min and transferred to 1.5‐ml tubes. The cells were then lysed in sonication buffer (50 mM Tris‐HCl pH 8.0, 10 mM EDTA, 1% SDS, 1 mM PMSF, cocktail) and sonicated using a Bioruptor (Diagenode). The supernatant was diluted 1:10 in RIPA buffer (10 mM Tris‐HCl pH 8.0, 1 mM EDTA, 140 mM NaCl, 1% Triton X‐100, 0.1% SDS, 0.1% Na‐deoxycholate, cocktail) and incubated with Dynabeads M‐280 Streptavidin (11206D, Thermo Fisher Scientific) for affinity purification. The beads were washed once with low salt buffer (150 mM NaCl, 0.1% SDS, 1% Triton X‐100, 2 mM EDTA, 20 mM Tris‐HCl pH 8.0), once with high salt buffer (500 mM NaCl, 0.1% SDS, 1% Triton X‐100, 2 mM EDTA, 20 mM Tris‐HCl pH 8.0), once with LiCl buffer (250 mM LiCl, 1% NP‐40, 1% Na‐deoxycholate, 1 mM EDTA, 10 mM Tris‐HCl pH 8.0), and twice with TE buffer (10 mM Tris‐HCl pH 8.0, 1 mM EDTA). Then, the beads were collected into new Eppendorf tubes with TE buffer and incubated on a mixing block at 65°C overnight to reverse the formaldehyde cross‐links. The supernatant was incubated with 200 ng/μl RNase A at 37°C for 1 hr and then 200 ng/μl proteinase K at 55°C for 2 hr. The DNA was extracted with phenol and precipitated with ethanol using 30 μg glycogen (10901393001, Roche) as the carrier. Finally, the DNA pellet was dissolved in TE buffer.

### 4.9 ChIP‐seq and RNA‐seq analysis

ChIP‐seq and RNA‐seq libraries were prepared with an NEBNext^®^ Ultra™ DNA (or RNA) Library Prep Kit for Illumina (NEB), and paired‐end sequencing (PE150) was performed on an Illumina HiSeq™ 3000 platform at Guangzhou RiboBio Co., Ltd. Adapters and low‐quality reads were removed with standard quality control measures. For the ChIP‐seq analysis, the data were first aligned to the hg38 human genome assembly using Bowtie (v2.2.5) with the settings “‐‐very‐sensitive.” Low‐quality mapped reads were removed using SAMtools (v1.3.1) with the settings “‐q 30.” Peaks were called using MACS2 (v2.1.0) with the settings “‐‐keep‐dup 1 ‐q 0.05,” and further annotated by HOMER (v4.10). For RNA‐seq, the reads were aligned with HISAT2 (v2.1.0). Then, the aligned read numbers mapped to each gene were counted by HTSeq (v0. 6.0) and presented as the reads per kilobase of transcript per million mapped reads (RPKMs). Differentially expressed genes were determined by an adjusted *p*‐value threshold of <.05 using DESeq software.

### 4.10 Statistical analyses

Statistical analyses were performed in software GraphPad Prism 5 using a two‐tailed *t* test. Statistical significance was considered as *p < .05, **p < .01, ***p < .001. The data represent the means ± *SEM* of three independent experiments.

## CONFLICT OF INTEREST

The authors declare no conflict of interest.

## AUTHORS' CONTRIBUTION

M.W. and L.W. conducted the experiments; M.Q., X.T., Z.L., Y.L., Y.A., Y.H., and Y.M. provided technical support and analyzed data; L.S., L.P., and X.C. contributed to resources; Z.W., B.Q., and B.L. contributed to reviewing and editing the manuscript; M.W. and B.L. designed this study and wrote the manuscript.

## Supporting information

Supplementary Material

Supplementary File S7‐S9

Supplementary File S1‐S5

Supplementary File S6

## Data Availability

The raw data and processed ChIP‐seq and RNA‐seq data derived from this study are deposited in the NCBI GEO public database with accession number GSE137085.

## References

[acel13147-bib-0001] Ao, Y. , Zhang, J. , Liu, Z. , Qian, M. , Li, Y. , Wu, Z. , … Wang, Z. (2019). Lamin A buffers CK2 kinase activity to modulate aging in a progeria mouse model. Science Advances, 5(3), eaav5078 10.1126/sciadv.aav5078 30906869 PMC6426468

[acel13147-bib-0002] Aoto, T. , Saitoh, N. , Ichimura, T. , Niwa, H. , & Nakao, M. (2006). Nuclear and chromatin reorganization in the MHC‐Oct3/4 locus at developmental phases of embryonic stem cell differentiation. Developmental Biology, 298(2), 354–367. 10.1016/j.ydbio.2006.04.450 16950240

[acel13147-bib-0003] Boichuk, S. , Hu, L. , Makielski, K. , Pandolfi, P. P. , & Gjoerup, O. V. (2011). Functional connection between Rad51 and PML in homology‐directed repair. PLoS ONE, 6(10), e25814 10.1371/journal.pone.0025814 21998700 PMC3187806

[acel13147-bib-0004] Capell, B. C. , Erdos, M. R. , Madigan, J. P. , Fiordalisi, J. J. , Varga, R. , Conneely, K. N. , … Collins, F. S. (2005). Inhibiting farnesylation of progerin prevents the characteristic nuclear blebbing of Hutchinson‐Gilford progeria syndrome. Proceedings of the National Academy of Sciences of the United States of America, 102(36), 12879–12884. 10.1073/pnas.0506001102 16129833 PMC1200293

[acel13147-bib-0005] Chang, H. R. , Munkhjargal, A. , Kim, M.‐J. , Park, S. Y. , Jung, E. , Ryu, J.‐H. , … Kim, Y. (2018). The functional roles of PML nuclear bodies in genome maintenance. Mutation Research, 809, 99–107. 10.1016/j.mrfmmm.2017.05.002 28521962

[acel13147-bib-0006] Ching, R. W. , Ahmed, K. , Boutros, P. C. , Penn, L. Z. , & Bazett‐Jones, D. P. (2013). Identifying gene locus associations with promyelocytic leukemia nuclear bodies using immuno‐TRAP. Journal of Cell Biology, 201(2), 325–335. 10.1083/jcb.201211097 23589495 PMC3628506

[acel13147-bib-0007] Ching, R. W. , Dellaire, G. , Eskiw, C. H. , & Bazett‐Jones, D. P. (2005). PML bodies: A meeting place for genomic loci? Journal of Cell Science, 118(Pt 5), 847–854. 10.1242/jcs.01700 15731002

[acel13147-bib-0008] Columbaro, M. , Capanni, C. , Mattioli, E. , Novelli, G. , Parnaik, V. K. , Squarzoni, S. , … Lattanzi, G. (2005). Rescue of heterochromatin organization in Hutchinson‐Gilford progeria by drug treatment. Cellular and Molecular Life Sciences, 62(22), 2669–2678. 10.1007/s00018-005-5318-6 16261260 PMC2773834

[acel13147-bib-0009] Condemine, W. , Takahashi, Y. , Zhu, J. , Puvion‐Dutilleul, F. , Guegan, S. , Janin, A. , & de The, H. (2006). Characterization of endogenous human promyelocytic leukemia isoforms. Cancer Research, 66(12), 6192–6198. 10.1158/0008-5472.CAN-05-3792 16778193

[acel13147-bib-0010] Delbarre, E. , Ivanauskiene, K. , Spirkoski, J. , Shah, A. , Vekterud, K. , Moskaug, J. Ø. , … Collas, P. (2017). PML protein organizes heterochromatin domains where it regulates histone H3.3 deposition by ATRX/DAXX. Genome Research, 27(6), 913–921. 10.1101/gr.215830.116 28341773 PMC5453325

[acel13147-bib-0011] Geng, Y. , Monajembashi, S. , Shao, A. , Cui, D. I. , He, W. , Chen, Z. , … Tang, J. (2012). Contribution of the C‐terminal regions of promyelocytic leukemia protein (PML) isoforms II and V to PML nuclear body formation. Journal of Biological Chemistry, 287(36), 30729–30742. 10.1074/jbc.M112.374769 22773875 PMC3436317

[acel13147-bib-0012] Gonzalo, S. , Kreienkamp, R. , & Askjaer, P. (2017). Hutchinson‐Gilford progeria syndrome: A premature aging disease caused by LMNA gene mutations. Ageing Research Reviews, 33, 18–29. 10.1016/j.arr.2016.06.007 27374873 PMC5195863

[acel13147-bib-0013] Gorgoulis, V. , Adams, P. D. , Alimonti, A. , Bennett, D. C. , Bischof, O. , Bishop, C. , … Demaria, M. (2019). Cellular senescence: Defining a path forward. Cell, 179(4), 813–827. 10.1016/j.cell.2019.10.005 31675495

[acel13147-bib-0014] Hamczyk, M. R. , del Campo, L. , & Andres, V. (2018). Aging in the cardiovascular system: Lessons from Hutchinson‐Gilford progeria syndrome. Annual Review of Physiology, 80, 27–48. 10.1146/annurev-physiol-021317-121454 28934587

[acel13147-bib-0015] Harhouri, K. , Navarro, C. , Depetris, D. , Mattei, M. G. , Nissan, X. , Cau, P. , … Levy, N. (2017). MG132‐induced progerin clearance is mediated by autophagy activation and splicing regulation. EMBO Molecular Medicine, 9(9), 1294–1313. 10.15252/emmm.201607315 28674081 PMC5582415

[acel13147-bib-0016] Hernandez‐Segura, A. , Nehme, J. , & Demaria, M. (2018). Hallmarks of cellular senescence. Trends in Cell Biology, 28(6), 436–453. 10.1016/j.tcb.2018.02.001 29477613

[acel13147-bib-0017] Hsu, K. S. , & Kao, H. Y. (2018). PML: Regulation and multifaceted function beyond tumor suppression. Cell & Bioscience, 8, 5 10.1186/s13578-018-0204-8 29416846 PMC5785837

[acel13147-bib-0018] Jul‐Larsen, A. , Grudic, A. , Bjerkvig, R. , & Boe, S. O. (2010). Subcellular distribution of nuclear import‐defective isoforms of the promyelocytic leukemia protein. BMC Molecular Biology, 11, 89 10.1186/1471-2199-11-89 21092142 PMC2998510

[acel13147-bib-0019] Kubben, N. , Zhang, W. , Wang, L. , Voss, T. C. , Yang, J. , Qu, J. , … Misteli, T. (2016). Repression of the antioxidant NRF2 pathway in premature aging. Cell, 165(6), 1361–1374. 10.1016/j.cell.2016.05.017 27259148 PMC4893198

[acel13147-bib-0020] Lallemand‐Breitenbach, V. , & de The, H. (2010). PML nuclear bodies. Cold Spring Harbor Perspectives in Biology, 2(5), a000661 10.1101/cshperspect.a000661 20452955 PMC2857171

[acel13147-bib-0021] Lallemand‐Breitenbach, V. , & de The, H. (2018). PML nuclear bodies: From architecture to function. Current Opinion in Cell Biology, 52, 154–161. 10.1016/j.ceb.2018.03.011 29723661

[acel13147-bib-0022] Lewis, P. W. , Elsaesser, S. J. , Noh, K. M. , Stadler, S. C. , & Allis, C. D. (2010). Daxx is an H3.3‐specific histone chaperone and cooperates with ATRX in replication‐independent chromatin assembly at telomeres. Proceedings of the National Academy of Sciences of the United States of America, 107(32), 14075–14080. 10.1073/pnas.1008850107 20651253 PMC2922592

[acel13147-bib-0023] Li, C. , Peng, Q. , Wan, X. , Sun, H. , & Tang, J. (2017). C‐terminal motifs in promyelocytic leukemia protein isoforms critically regulate PML nuclear body formation. Journal of Cell Science, 130(20), 3496–3506. 10.1242/jcs.202879 28851805

[acel13147-bib-0024] Liu, B. , Wang, J. , Chan, K. M. , Tjia, W. M. , Deng, W. , Guan, X. , … Zhou, Z. (2005). Genomic instability in laminopathy‐based premature aging. Nature Medicine, 11(7), 780–785. 10.1038/nm1266 15980864

[acel13147-bib-0025] Liu, G.‐H. , Barkho, B. Z. , Ruiz, S. , Diep, D. , Qu, J. , Yang, S.‐L. , … Belmonte, J. C. I. (2011). Recapitulation of premature ageing with iPSCs from Hutchinson‐Gilford progeria syndrome. Nature, 472(7342), 221–225. 10.1038/nature09879 21346760 PMC3088088

[acel13147-bib-0026] Mattioli, E. , Andrenacci, D. , Garofalo, C. , Prencipe, S. , Scotlandi, K. , Remondini, D. , … Lattanzi, G. (2018). Altered modulation of lamin A/C‐HDAC2 interaction and p21 expression during oxidative stress response in HGPS. Aging Cell, 17(5), e12824 10.1111/acel.12824 30109767 PMC6156291

[acel13147-bib-0027] Nisole, S. , Maroui, M. A. , Mascle, X. H. , Aubry, M. , & Chelbi‐Alix, M. K. (2013). Differential roles of PML isoforms. Frontiers in Oncology, 3, 125 10.3389/fonc.2013.00125 23734343 PMC3660695

[acel13147-bib-0028] Ohsaki, Y. , Kawai, T. , Yoshikawa, Y. , Cheng, J. , Jokitalo, E. , & Fujimoto, T. (2016). PML isoform II plays a critical role in nuclear lipid droplet formation. Journal of Cell Biology, 212(1), 29–38. 10.1083/jcb.201507122 26728854 PMC4700481

[acel13147-bib-0029] Salsman, J. , Stathakis, A. , Parker, E. , Chung, D. , Anthes, L. E. , Koskowich, K. L. , … Dellaire, G. (2017). PML nuclear bodies contribute to the basal expression of the mTOR inhibitor DDIT4. Scientific Reports, 7, 45038 10.1038/srep45038 28332630 PMC5362932

[acel13147-bib-0030] Shastrula, P. K. , Sierra, I. , Deng, Z. , Keeney, F. , Hayden, J. E. , Lieberman, P. M. , & Janicki, S. M. (2019). PML is recruited to heterochromatin during S phase and represses DAXX‐mediated histone H3.3 chromatin assembly. Journal of Cell Science, 132(6), jcs220970 10.1242/jcs.220970 30796101 PMC6451418

[acel13147-bib-0031] Toth, J. I. , Yang, S. H. , Qiao, X. , Beigneux, A. P. , Gelb, M. H. , Moulson, C. L. , … Fong, L. G. (2005). Blocking protein farnesyltransferase improves nuclear shape in fibroblasts from humans with progeroid syndromes. Proceedings of the National Academy of Sciences of the United States of America, 102(36), 12873–12878. 10.1073/pnas.0505767102 16129834 PMC1193538

[acel13147-bib-0032] Ulbricht, T. , Alzrigat, M. , Horch, A. , Reuter, N. , von Mikecz, A. , Steimle, V. , … Hemmerich, P. (2012). PML promotes MHC class II gene expression by stabilizing the class II transactivator. Journal of Cell Biology, 199(1), 49–63. 10.1083/jcb.201112015 23007646 PMC3461510

[acel13147-bib-0033] Van Damme, E. , Laukens, K. , Dang, T. H. , & Van Ostade, X. (2010). A manually curated network of the PML nuclear body interactome reveals an important role for PML‐NBs in SUMOylation dynamics. International Journal of Biological Sciences, 6(1), 51–67. 20087442 10.7150/ijbs.6.51 PMC2808052

[acel13147-bib-0034] Varela, I. , Pereira, S. , Ugalde, A. P. , Navarro, C. L. , Suárez, M. F. , Cau, P. , … López‐Otín, C. (2008). Combined treatment with statins and aminobisphosphonates extends longevity in a mouse model of human premature aging. Nature Medicine, 14(7), 767–772. 10.1038/nm1786 18587406

[acel13147-bib-0035] Voisset, E. , Moravcsik, E. , Stratford, E. W. , Jaye, A. , Palgrave, C. J. , Hills, R. K. , … Grimwade, D. (2018). Pml nuclear body disruption cooperates in APL pathogenesis and impairs DNA damage repair pathways in mice. Blood, 131(6), 636–648. 10.1182/blood-2017-07-794784 29191918 PMC5805489

[acel13147-bib-0036] Yeung, P. L. , Denissova, N. G. , Nasello, C. , Hakhverdyan, Z. , Chen, J. D. , & Brenneman, M. A. (2012). Promyelocytic leukemia nuclear bodies support a late step in DNA double‐strand break repair by homologous recombination. Journal of Cellular Biochemistry, 113(5), 1787–1799. 10.1002/jcb.24050 22213200 PMC3337353

[acel13147-bib-0037] Zhong, S. , Hu, P. , Ye, T. Z. , Stan, R. , Ellis, N. A. , & Pandolfi, P. P. (1999). A role for PML and the nuclear body in genomic stability. Oncogene, 18(56), 7941–7947. 10.1038/sj.onc.1203367 10637504

[acel13147-bib-0038] Zhong, S. , Salomoni, P. , & Pandolfi, P. P. (2000). The transcriptional role of PML and the nuclear body. Nature Cell Biology, 2(5), E85–90. 10.1038/35010583 10806494

