# Supplementary material for: PML2‐mediated thread‐like nuclear bodies mark late senescence in Hutchinson–Gilford progeria syndrome
Source: Aging Cell. 2020 Apr 29;19(6):e13147. doi: 10.1111/acel.13147 (PMC7294779; doi:10.1111/acel.13147)

# Supporting Data S1

***Experimental procedures***

**Transfection and virus infection**

Transfection in HeLa cells was performed using Lipofectamine3000 (L3000015, Thermo Fisher Scientific) following standard procedures. siRNA was transfected with DharmaFECT$^{TM}$ (T-2001, Thermo Fisher Scientific) in Opti-MEM medium and the mix was incubated with cells for 6 h. At 72 h after transfection, cells were harvested for RNA extraction or other analysis. The sequence information of siRNA was listed in Supplementary File S8. Lenti-virus pLVX and pKD were used in this study for gene expression. Virus was packaged by transfecting recombinant plasmids together with packaging plasmids psPAX2 and pMD2.G into HEK293T cells using Lipofectamine3000$^{-}$, harvested at 48 h after transfection and filtered using 0.22 μM membrane filters before further use. Virus diluted at 1:1 with fresh medium containing 8 mg/L of polybrene were added to cells and cultured for 10 h. Puromycin was used to screen out successfully infected cells.

**X-ray irradiation**

Cells plated on coverslips were exposed to 2 Gy of irradiation in an X-ray Biological Irradiator (RS 2000, Rad Source Technologies). Cells were harvested at indicated time points after irradiation and prepared for further analysis.

**Immunofluorescence microscopy**

Fibroblasts plated on coverslips were fixed in 4% paraformaldehyde (PFA) for 5 min and permeabilized with 0.2% Triton X-100 for 5 min. After blocking with 3% BSA for 1 h, cells were incubated with primary antibodies overnight at 4 °C, and then incubated with fluorescence conjugated secondary antibodies for another 1 h at room temperature. HeLa cells expressing GFP-lamins and mCherry-PMLs were fixed with 4% PFA for 3 min and then stained with DAPI before preserved in mounting medium. Photos were collected using LSM 800 confocal microscope (Carl Zeiss) and Axio Vert.A1 microscope (Carl Zeiss). Primary antibodies used in this study are as follow: Lamin B1 (ab16048, Abcam), H3K27me3 (07-449, Millipore), LAP2β (ab185718, Abcam), γH2AX (05-636, Millipore), Ki-67 (M7240, Dako), Progerin (sc-81611, Santa Cruz), Lamin A/C (sc-7292, Santa Cruz), Rad51 (ab63801, Abcam), PML mouse antibody (sc-966, Santa Cruz) and rabbit antibody (ab190899, Abcam).

**Immunoprecipitation and Western blotting**

Cells were collected in lysis buffer (20 mM Tris-HCl pH 7.4, 300 mM NaCl, 0.2% NP-40, 10% glycerol, 1 mM EDTA, 1 mM PMSF and protease inhibitor cocktail) and lysed by sonication. After centrifugation at 13,000 rpm for 20 min at 4 °C, supernatant was incubated with anti GFP antibodies or PML antibodies conjugated Dynabeads® Protein A/G (10001D, 10003D, Life technologies) or anti-FLAG® M2 magnetic beads (M8823, Sigma) at 4 °C on a rotator for 3 h. Subsequently, beads were rinsed with lysis buffer for 3 times and then boiled in loading buffer for 5 min. Samples were subjected to SDS-PAGE electrophoresis and transferred to PVDF membrane. After blocking with 5% fat-free milk in TBS-T buffer (TBS buffer, 10 mM Tris-HCl pH 7.5, 150 mM NaCl, 0.1% Tween-20), the membrane was incubated with corresponding antibodies: FLAG M2 (F1804, Sigma), GFP (sc-9996, Santa Cruz), Lamin A/C (sc-20681, Santa Cruz), PML (NB100-59787, Novus Biology), Actin (A2066, Sigma) and Progerin (sc-81611, Santa Cruz) for 1 h at room temperature or overnight at 4 °C. Then secondary antibodies conjugated HRP were added and incubated for 1 h before detection using ECL solution.

**Senescence associated β-galactosidase (SA-β-gal) staining**

Cells cultured in a 12-well plate were fixed using the fixative solution provided by the senescence β-galactosidase staining kit (9860, Cell Signaling), and β-gal staining was performed according to manufacturer's protocol. Each well was incubated with 800 μL of freshly prepared staining solution at 37 °C in a dry incubator for 12 h. For accurate and convenient counting of cell number, nuclei were counterstained with DAPI after β-gal staining. For co-staining of PML, ~50% confluence of cells cultured on glass coverslip were fixed for 10 min. After SA-β-gal staining, cells were permeabilized with 0.2% Triton X-100 for 10 min, and further immunofluorescence staining was performed as described above.

**RNA extraction and qRT-PCR**

Cells were lysed with TRIzol® (15596026, Thermo Fisher Scientific) and total RNA was extracted according to the manufacturer's instruction. 2 μg of RNA was used for cDNA synthesis via reverse transcription PCR (TRT-101, TOYOBO). Quantitative PCR was performed on Applied Biosystems 7500 using FastStart Universal SYBR Green Master (04913914001, Roche). Relative gene expression was quantified by normalizing to *Actb* values. Primers for qRT-PCR were listed in Supplementary File S9.

***Supplementary Figure Legends***

**Supplementary Figure S1** Thread-like PML NBs exist in HGPS cell lines and late-passage NHDF cells. (a) NHDF cells and HGPS cell lines (HG122, HG143, HG155 and HG169) were passaged every 3 days and population doublings (PDs) were analyzed. (b) PML and Lamin A/C IF staining in HGPS cell lines. Scale bar, 10 μm. (c) PML and Lamin A/C IF staining in late-passage NHDF (P50) and HGPS (P32) cells. Arrows indicate cells with thread-like PML NBs. Scale bar, 20 μm. (d) Ki-67 and SA-β-gal staining analysis of NHDF cells (at Passage 50) with or without thread-like PML NBs, $^{**}P < 0.01$, $^{***}P < 0.001$.

**Supplementary Figure S2** Progerin interacts with PML isoform 2 specifically. (a) Flag-PML isoforms 1~6 were co-transfected with GFP, -Lamin A or -Progerin in 293T cells for 18 h, and then cells were lysed for immunoprecipitation (IP) assay using the GFP antibody. (b) Determination of knockdown efficacy of siRNAs specifically targeting to pan-PML and PML isoforms. Quantitative RT-PCR analysis of mRNA expression of pan-PML and PML isoforms after siRNA treatment.

**Supplementary Figure S3** Exogenous expression of PML2 inhibits cell proliferation and perturbs DNA repair. (a) Ki-67 was stained in NHDF and HGPS cells expressing GFP, GFP-PML1 or GFP-PML2. Scale bar, 20 μm. (b) γH2AX IF staining in NHDF and HGPS cells expressing GFP, GFP-PML1 or GFP-PML2. Scale bar, 10 μm. (c) Time-course analysis of γH2AX foci in NHDF and HGPS cells expressing GFP, GFP-PML1 or GFP-PML2 after 2 Gy of X-ray irradiation. $^{**}P < 0.01$; compared to HGPS GFP-PML1 group. (d) At 24 h after 2 Gy of X-ray irradiation, γH2AX was stained in NHDF and HGPS cells expressing GFP, GFP-PML1 or GFP-PML2. Scale bar, 20 μm.

**Supplementary Figure S4** PML NBs associate with gene promoters. (a) Immunofluorescence staining analysis of PML (show in green) and biotin-tyramide deposition using streptavidin conjugated with Alexa 568 (show in red) in NHDF cells to determine the quality of immuno-TRAP assay. (b) Genome view of peak signals situated in the promoter region of genes such as *CDK4*, *HRAS*, *STAT3*, *KAT6A*, *PER1*, *PER2*, *DDIT4* and *FOXO3*. The tracks in blue were shown as the "Input" control. chr, chromosome.

***Supplementary Files***

**Supplementary File S1** Total 1,972 of differential peaks were identified by immuno-TRAP and ChIP-seq

**Supplementary File S2** Total 1,712 of PML NBs-associated genes were identified from 1972 peaks

**Supplementary File S3** GO and KEGG analysis of 1,712 PML NBs-associated genes

**Supplementary File S4** RNA-seq of gene expression

**Supplementary File S5** Total 858 of PML NBs-associated genes were differentially expressed in PML siRNA (NFsi) treated cells compared to control siRNA (NFcon) treated cells

**Supplementary File S6** Total 855 of 1,712 PML NBs-associated genes were aberrantly expressed in HGPS cells (HGcon) compared to NHDF cells (NFcon). PML2 siRNA (HGI2si) and FTI (HGFTI) treatment in HGPS cells significantly alleviated the expression of 365 and 343 genes respectively ($P < 0.05$). Total 230 genes were overlapped between the HGI2si and HGFTI group.

**Supplementary File S7** The list of senescence-associated genes identified from 230 overlapped genes between the HGI2si and HGFTI group.

**Supplementary File S8** Sequence information of siRNA for pan-PML and PML isoforms

**Supplementary File S9** Sequence information of primers for q-PCR

# Supplementary Figures

## Figure S1

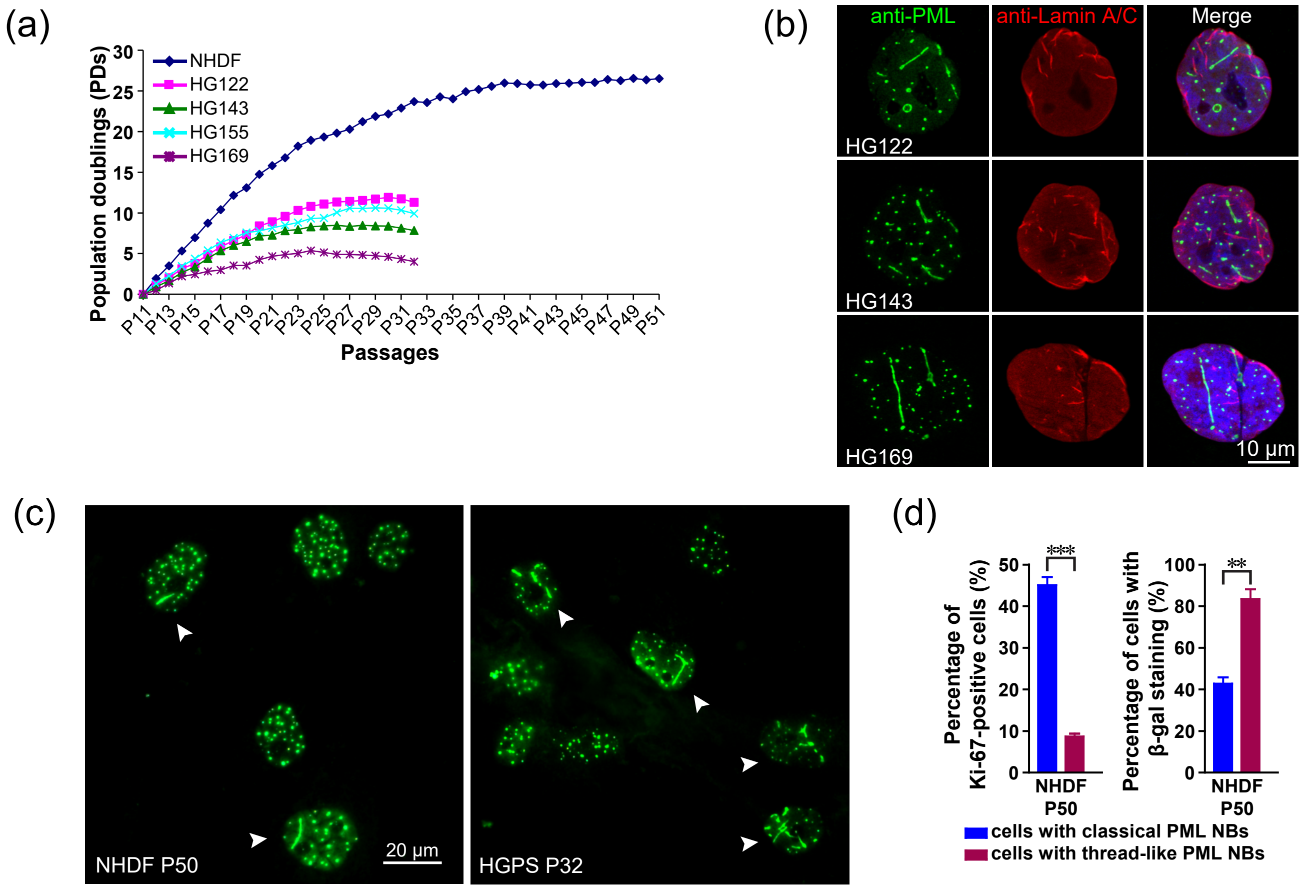


## Figure S2

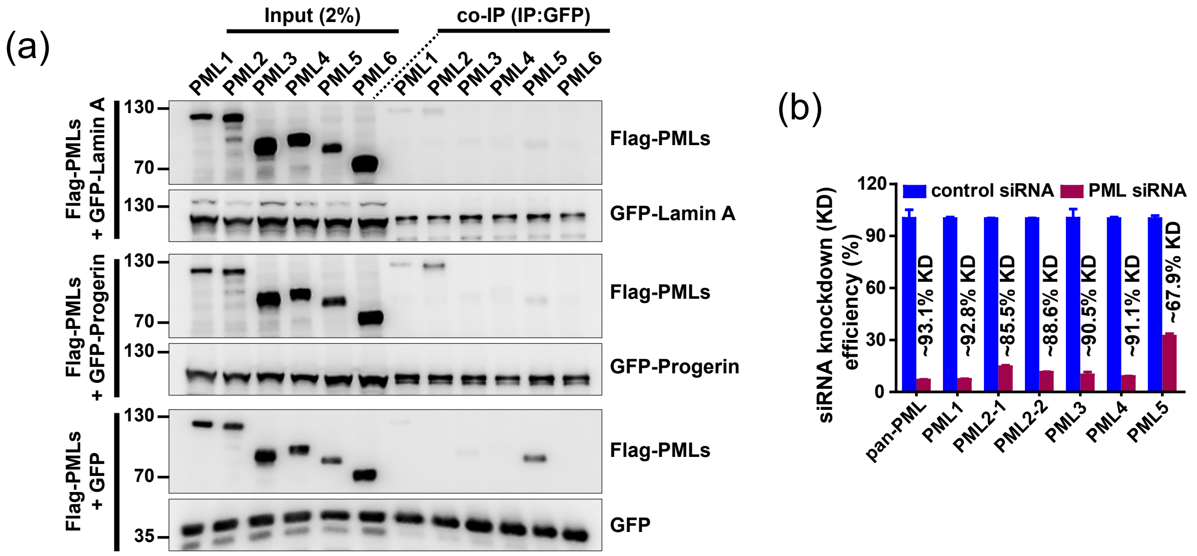

# Figure S3

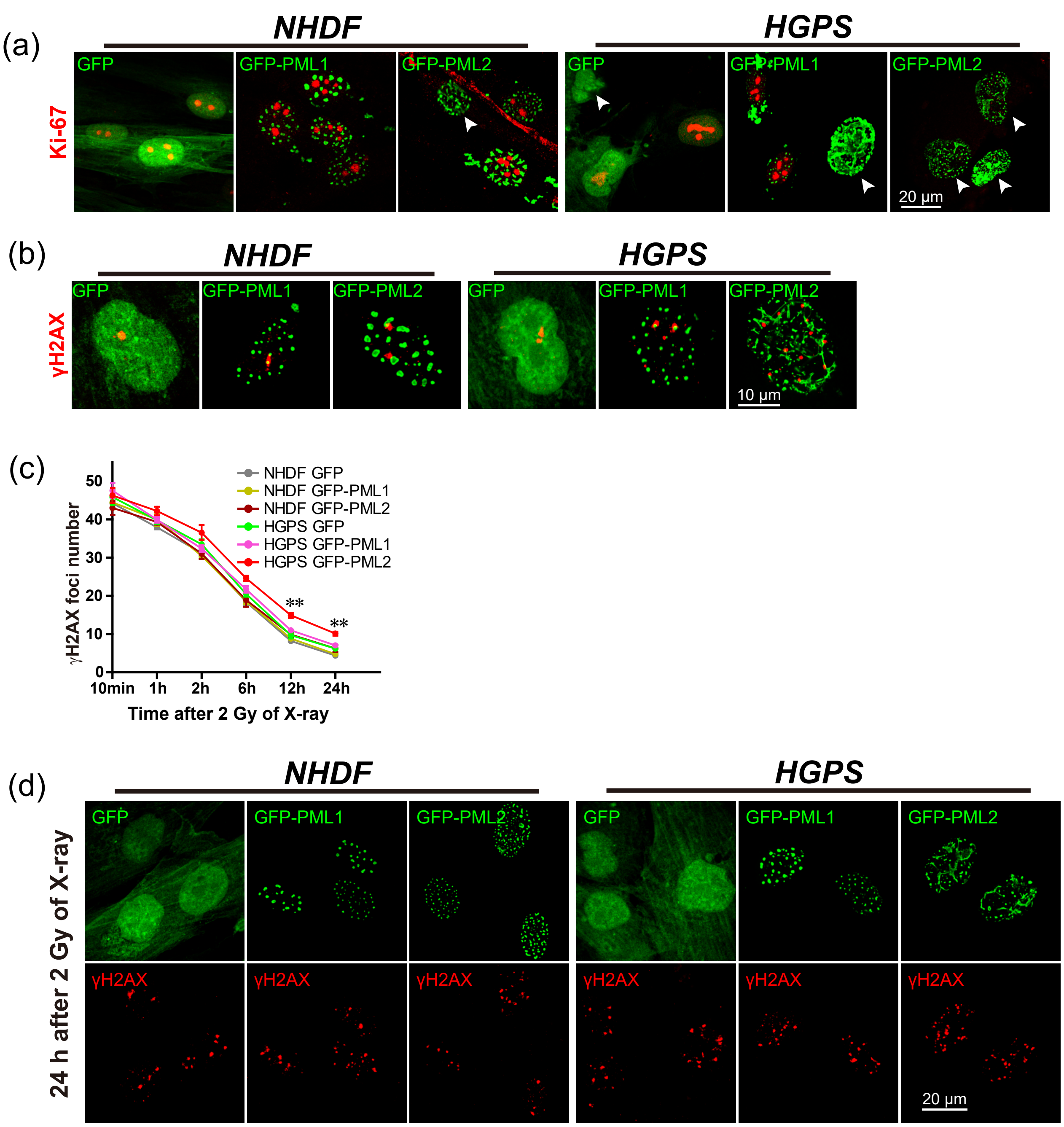


# Figure S4

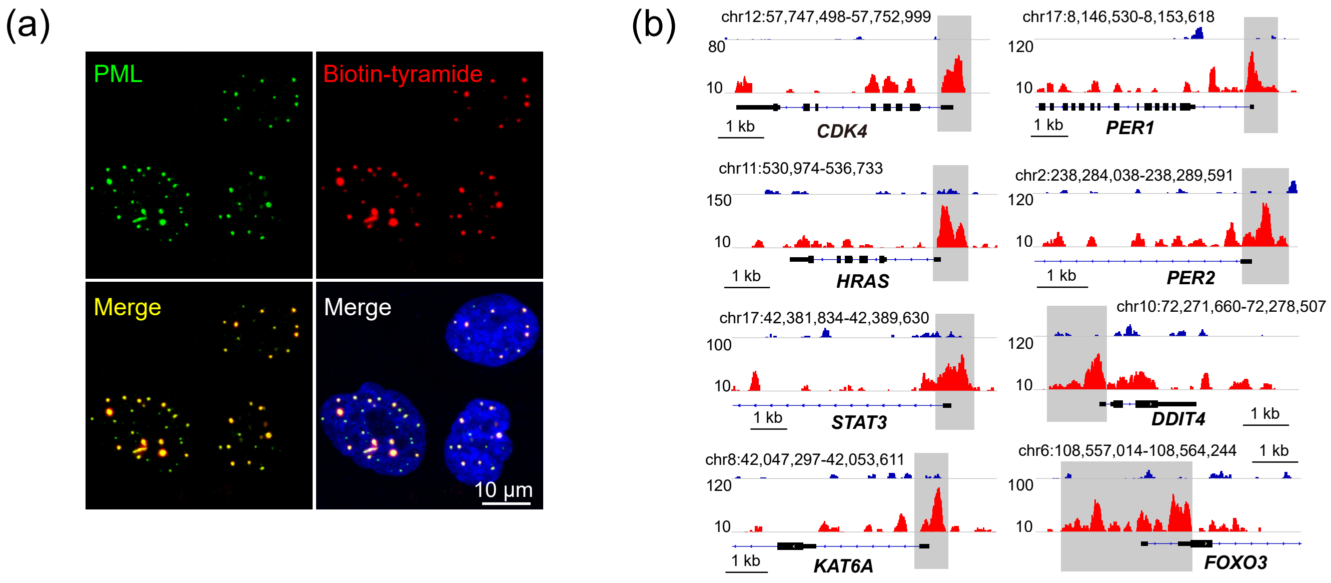

Supplement: Supplementary file 1 — Supplementary Material [file ACEL-19-e13147-s001.pdf]
